# Intra-islet GLP-1, but not CCK, is necessary for β-cell function in mouse and human islets

**DOI:** 10.1038/s41598-020-59799-2

**Published:** 2020-02-18

**Authors:** Arnaldo Henrique de Souza, Jiayin Tang, Amanjot Kaur Yadev, Samuel T. Saghafi, Carly R. Kibbe, Amelia K. Linnemann, Matthew J. Merrins, Dawn Belt Davis

**Affiliations:** 1 Department of Medicine, Division of Endocrinology, Diabetes, and Metabolism, University of Wisconsin-Madison, Madison, WI USA; 2 Department of Human Biology, University of Wisconsin-Green Bay, Green Bay, WI USA; 3 Department of Pediatrics and Center for Diabetes and Metabolic Diseases, Indiana University School of Medicine, Indianapolis, Indiana USA; 4 William S. Middleton Memorial Veterans Hospital, Madison, WI USA

**Keywords:** Homeostasis, Diabetes, Obesity

## Abstract

Glucagon-like peptide 1 (GLP-1) and cholecystokinin (CCK) are gut-derived peptide hormones known to play important roles in the regulation of gastrointestinal motility and secretion, appetite, and food intake. We have previously demonstrated that both GLP-1 and CCK are produced in the endocrine pancreas of obese mice. Interestingly, while GLP-1 is well known to stimulate insulin secretion by the pancreatic β-cells, direct evidence of CCK promoting insulin release in human islets remains to be determined. Here, we tested whether islet-derived GLP-1 or CCK is necessary for the full stimulation of insulin secretion. We confirm that mouse pancreatic islets secrete GLP-1 and CCK, but only GLP-1 acts locally within the islet to promote insulin release *ex vivo*. GLP-1 is exclusively produced in approximately 50% of α-cells in lean mouse islets and 70% of α-cells in human islets, suggesting a paracrine α to β-cell signaling through the β-cell GLP-1 receptor. Additionally, we provide evidence that islet CCK expression is regulated by glucose, but its receptor signaling is not required during glucose-stimulated insulin secretion (GSIS). We also see no increase in GSIS in response to CCK peptides. Importantly, all these findings were confirmed in islets from non-diabetic human donors. In summary, our data suggest no direct role for CCK in stimulating insulin secretion and highlight the critical role of intra-islet GLP-1 signaling in the regulation of human β-cell function.

## Introduction

The precise control of blood glucose is dependent on the islets of Langerhans located within the pancreas. Pancreatic islets are complex structures consisting of several types of cells, including insulin-producing β-cells, glucagon-producing α-cells, and somatostatin-producing α-cells. After feeding, nutrients stimulate insulin release by the β-cell and the circulating hormone acts on peripheral tissues lowering blood glucose. The full stimulation of insulin secretion after food intake depends on the incretin effect of gut-derived peptide hormones and paracrine signaling within the islet^1^.

We and others discovered that two classic gut hormones, glucagon-like peptide 1 (GLP-1) and cholecystokinin (CCK), are also produced by pancreatic islets^2–5^. GLP-1 is a peptide hormone mainly secreted by intestinal L-cells and is known to decrease blood glucose levels by enhancing β-cell insulin secretion^6^. CCK is a peptide hormone mainly secreted by intestinal I-cells and is involved in the digestion of nutrients^7^ and regulation of food intake^8^. The production of both GLP-1 and CCK in the islet has been associated with β-cell expansion and survival in response to metabolic stress^5,9^. While each of these gut-derived hormones can contribute to improvements in glucose homeostasis^10,11^, it is unclear whether their local production in the islet contributes to β-cell function. Moreover, it is unknown whether CCK stimulates insulin release in human islets. Here, we describe experiments with GLP-1 and CCK receptor antagonists in isolated mouse and human islets to assess β-cell function *ex vivo*. Our data demonstrate that pancreatic CCK is not required for glucose-stimulated insulin secretion (GSIS) and CCK peptide does not potentiate GSIS. Furthermore, we demonstrate that intra-islet GLP-1 signaling between α to β-cell is necessary for normal insulin secretion.

## Results

### Expression of pancreatic GLP-1 in mouse and human islets

**Figure 1:**
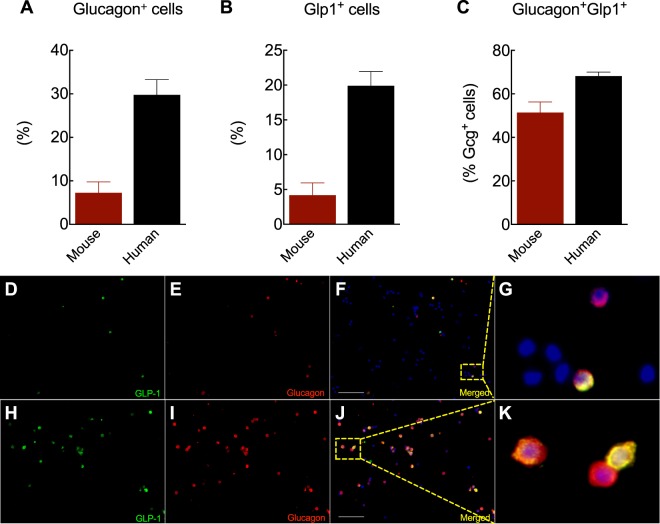
GLP-1 expression in pancreatic α-cells. (**A**) Percentage of glucagon-positive cells, (**B**) GLP1-positive cells, and (**C**) glucagon cells double positive for glucagon and GLP-1. Representative images of co-localization of GLP-1 (7–37) (green), glucagon (red) and DAPI (blue) in mouse (**D**–**G**) and human (**H**–**K**) islet cells. Scale bars, 200 µm. Data are mean ± SEM of 4 islet preparations.

Emerging evidence supports the production of GLP-1 in pancreatic islets^3,4,12,13^. Prohormone convertase 1/3 (PC1/3), the enzyme responsible for GLP-1 cleavage from a proglucagon precursor, has been detected in rodent glucagon-producing cells, especially under β-cell stress conditions^2,14,15^. However, the number of α-cells producing GLP-1 in non-stressed conditions has generally been assumed to be relatively few. Here, we evaluate whether fully processed active GLP-1 can be detected in islets isolated from lean mice and non-diabetic human donors and quantify its expression patterns using an antibody specific for the processed and biologically active GLP-1 (7–36) amide form that does not cross-react with glucagon peptide^3^. The challenge for α-cell quantification in rodents includes the low number of α-cells in the islet (<10% in mouse islets). To overcome this problem, we dispersed islets into single-cells right before staining to maximize the number of α-cells counted in each experiment. As expected, mouse islets had fewer α-cells compared to humans (Fig. 1A). We detected active GLP-1 in both mouse and human islet cells (Fig. 1B). Quantification analysis shows that approximately 50% of glucagon-positive cells co-express GLP-1 in lean mice (Fig. 1C–G). Surprisingly, these bi-hormonal cells were near 70% in humans (Fig. 1C,H–K). Essentially all of the GLP-1 expressing cells were α-cells. The body mass index (BMI) of the donors of the human islets used ranged from 28.5–31.8 kg/m^2^ (islet preparations 5–8, Suppl. Table 1), consistent with our previous study in which islet GLP-1 secretion is increased in obesity^4^. Also, dispersed mouse islet cells had a similar expression pattern as intact islets (Suppl. Fig. 1), suggesting that acute islet dispersion did not affect either glucagon or GLP-1 expression. Together, our results suggest that GLP-1 is produced in a high percentage of α-cells, especially in human islets.

### Paracrine GLP-1 signaling is necessary for GSIS

**Figure 2:**
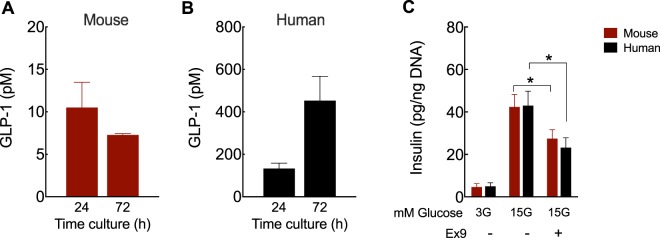
Paracrine GLP-1 signaling is required for *ex vivo* GSIS. GLP-1 (7–36 amide) and (7–37) released during the culture of mouse (**A**) and human (**B**) islets in RPMI containing 11 mM (**A**) and 8 mM glucose (**B**), respectively. (**C**) Insulin secreted from batches of 5 islets after 1 h incubation at 3 or 15 mM glucose (3 G and 15 G, respectively) or 15 G in the presence of 1 µM exendin-(9–39) (Ex9), a specific GLP-1R antagonist. Insulin values were normalized to the islet DNA content from each batch (total of 9–12 batches/condition). Significance was tested by two-way ANOVA and Bonferroni’s post-hoc test. Data are mean ± SEM of 3–4 islet preparations. ^*^P < 0.05.

To test whether α-cell-derived GLP-1 plays an important paracrine role in β-cell function, we first measured bioactive GLP-1 secreted into the islet media as detected by active GLP-1 ELISA. Human islets secreted over 10-fold more GLP-1 than mouse islets on a per islet basis (Fig. 2A,B), consistent with the higher number of GLP-1-positive cells observed in Fig. 1 as well as with a recent work comparing mouse vs. human pancreatic GLP-1^16^. We then tested whether paracrine GLP-1 signaling is necessary for normal glucose-stimulated insulin secretion by performing static GSIS studies in the presence of exendin-(9–39) (Ex9), a specific GLP-1 receptor (GLP-1R) antagonist^17^. We found that Ex-9 blunted GSIS in both mouse and human islets (Fig. 2C). Insulin content did not change significantly across all conditions and we found similar GSIS results when secretion was normalized to percentage of insulin content (data not shown). These results demonstrate that intra-islet GLP-1 signaling is necessary for GSIS in humans.

### CCK signaling in mouse and human islets

**Figure 3:**
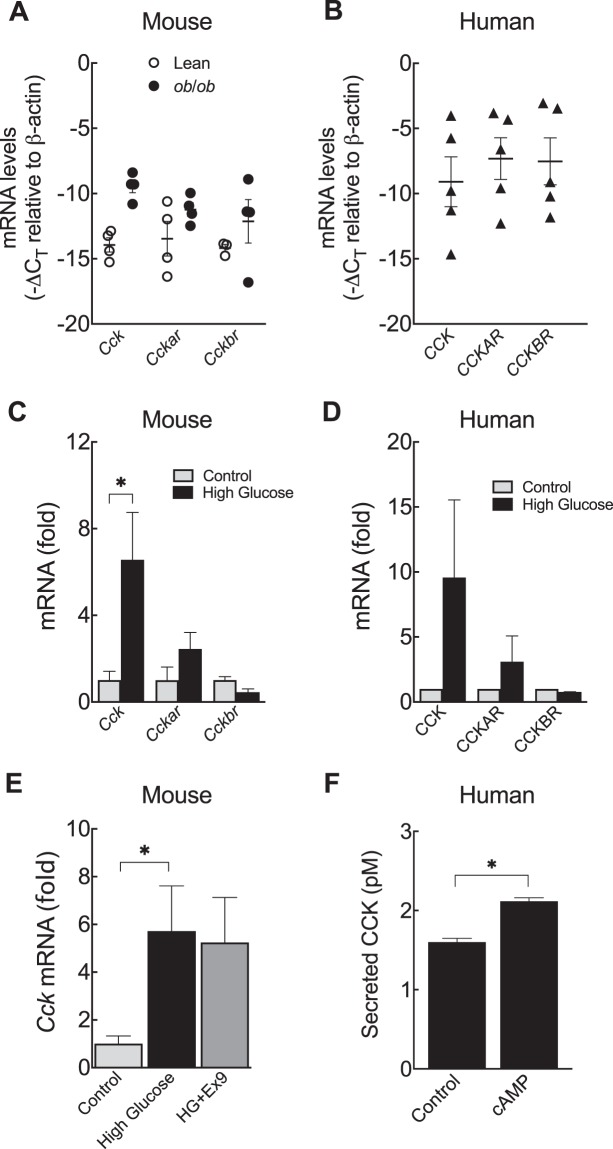
CCK gene expression is regulated by glucose. (**A**) mRNA levels of cholecystokinin (*Cck*), and CCK receptors (*Cckar* and *Cckbr*) in islets isolated from lean or obese mice. (**B**) *CCK*, *CCKAR*, and *CCKBR* in isolated human islets. mRNA levels in lean mouse (**C**) and human islets (**D**) after 24 h culture in high glucose (25 mM glucose). (**E**) *Cck* in lean mouse islets after 24 h culture in high glucose in presence of 200 nM exendin-(9–39) (HG + Ex9). (**F**) Sulfated cholecystokinin (CCK) released into the media during culture of human islets with 10 µM 8-CPT-cAMP. Significance was tested by Student’s t-test (**B**,**C**,**F**) and one-way ANOVA follow by Bonferroni’s post-hoc test (**D**). Data are mean ± SEM (n = 5 for mouse and n = 2 for human). ^*^P < 0.05.

CCK is another peptide hormone commonly expressed in neuroendocrine cells in the gut, yet rodent pancreatic islets also express and secrete CCK under conditions of metabolic stress such as obesity and insulin resistance^5,18,19^. Here, we confirmed our previous findings^5^ that *Cck* is up-regulated in leptin-deficient obese mouse islets (*ob*/*ob*) (Fig. 3A). Since CCK has two known receptors, CCKAR and CCKBR^20^, we then tested if a specific receptor signaling pathway is more relevant in the islet or changed by obesity. We found that islets from either lean or obese animals had relatively low mRNA levels of both *Cckar* and *Cckbr* (Fig. 3A). In human islets, we confirmed the presence of *CCK* and *CCKAR*^4^ and provide new evidence that *CCKBR* is also present (Fig. 3A). Unlike in mice, we found no significant correlation between *CCK*, *CCKAR*, or *CCKBR* expression and donor BMI among donors without diabetes (Suppl. Fig. 2).

Since insulin resistance and hyperglycemia are common correlates of obesity, we hypothesized that glucose may be regulating CCK expression in the islet. We have previously shown that the ability of cyclic AMP (cAMP) to stimulate CCK expression in INS-1 cells was enhanced in high glucose conditions^4^. We incubated islets in high concentrations of glucose and determined CCK expression by quantitative real-time PCR (qPCR). After 24 h exposure to high glucose, there was an approximately 7-fold increase in *Cck* mRNA levels in lean mouse islets (Fig. 3B). Interestingly, we found a similar stimulatory effect of glucose on CCK expression in response to glucose in isolated islets from two non-diabetic human donors (Fig. 3C). We saw no significant difference in CCKAR or CCKBR expression in response to glucose in either mouse or human islets (Fig. 3C,D). Because α-cells produce and release GLP-1^3,4,12,13^ (Figs. 1 and 2A,B) and GLP-1 can stimulate the production of CCK from β-cells^4^, we asked whether the induction of *Cck* by glucose is dependent on paracrine GLP-1 signaling. We found that glucose increased *Cck* by approximately 6-fold in mouse islets even when GLP-1R antagonist was present during the culture (Fig. 3E). Our previous work found that GLP-1 increases β-cell CCK mRNA expression and CCK peptide secretion through cAMP/CREB signaling in INS-1 cells^4^. Here we show that human islets also secrete active sulfated CCK peptide and that this secretion is enhanced by exposure to a cell-permeable cAMP analogue (Fig. 3F). Taken together, our results demonstrate that the effect of glucose on islet CCK expression is not GLP-1 receptor-dependent and further support the role of cAMP signaling in islet/β-cell CCK secretion even in human islets.

### Paracrine CCK signaling is not necessary for GSIS

**Figure 4:**
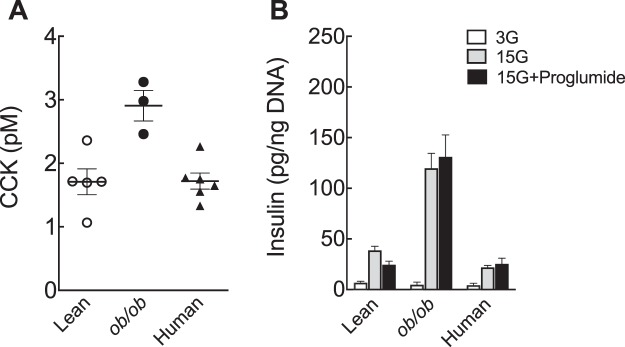
Paracrine CCK signaling is not required for *ex vivo* GSIS. (**A**) Sulfated cholecystokinin (CCK) released into the media during islet culture. (**B**) Insulin secreted from batches of 5 islets after 1 h incubation at 3 or 15 mM glucose (3 G and 15 G, respectively) or 15 G in the presence of 1 µM proglumide, a non-selective CCK receptor antagonist. Insulin values were normalized to the islet DNA content from each batch (total of 9–21 batches/condition). Significance was tested by two-way ANOVA and Bonferroni’s post-hoc test. Data are mean ± SEM of 3–7 islet. preparations.

Although it is not classified as an incretin hormone due to a general lack of effect on *in vivo* insulin secretion, CCK has been proposed to regulate insulin secretion in isolated rat islets^21^. We, therefore, hypothesized that, like pancreatic GLP-1, intra-islet CCK could also regulate β-cell insulin secretion. To test this, we performed *in vitro* and *ex vivo* studies in mouse and human islets. Here, we confirmed that CCK secretion is higher from *ob*/*ob* compared to lean mouse islets^5^ and show that non-diabetic human islets secrete small amounts of active, sulfated CCK peptide (Fig. 4A). Importantly, we used a radioimmunoassay specific for sulfated CCK peptides with no cross-reactivity to gastrin peptide (Alpco, based on assay developed in^22^). Since CCK has been proposed to stimulate insulin release in rodents^21^, we asked whether intra-islet CCK signaling is necessary for β-cell function. To answer this, we performed *ex vivo* GSIS in the presence of proglumide, a non-selective CCK receptor antagonist^23^. In contrast to Ex9 (Fig. 2C), we found that proglumide did not affect GSIS (Fig. 4B). This finding was consistent even in the islets from obese mice, which had both higher CCK and insulin release (Fig. 4A,B). Similarly, proglumide did not inhibit GSIS in human islets (Fig. 4B). Therefore, these results demonstrate that paracrine CCK signaling is not necessary for β-cell glucose stimulated insulin secretion.

### CCK does not regulate GSIS

**Figure 5:**
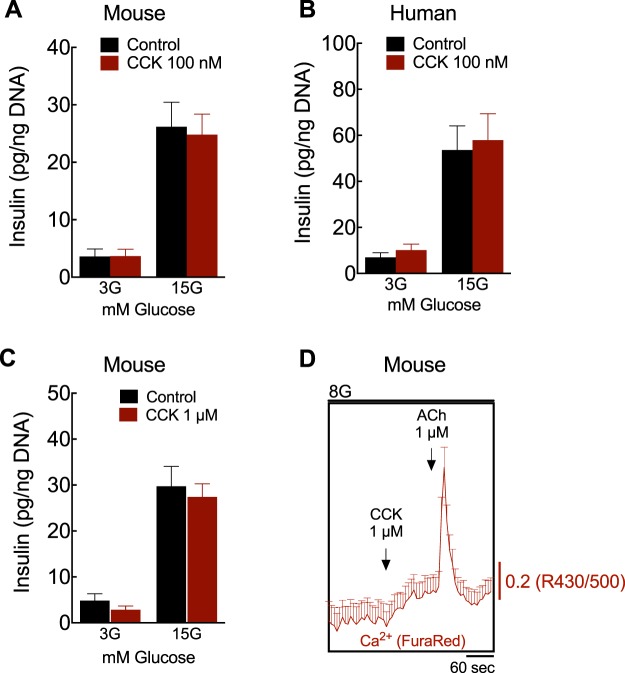
CCK does not stimulate *ex vivo* GSIS. Insulin secreted from (**A**,**C**) mouse and (**B**) human islets. Batches of 5 islets after 1 h incubation at 3 or 15 mM glucose (3 G and 15 G, respectively) in the presence of 100 nM (**A**,**B**) or 1 µM (**C**) sulfated pGlu-Gln-CCK-8 (CCK). Insulin values were normalized to DNA content from each batch (total of 9–12 batches/condition). (**C**) Cytosolic calcium (Ca^2+^) levels measured in mouse islet β-cells at 8 mM glucose (8 G) stimulated with 1 µM sulfated pGlu-Gln-CCK-8 (CCK) and 1 µM acetylcholine (ACh). Significance was tested by two-way ANOVA and Bonferroni’s post-hoc test. Data are mean ± SEM of 3–4 islet preparations.

The small amount of CCK released from the islet could be a reason for the lack of effect on β-cell function in a paracrine manner. However, in humans, the ability of CCK to stimulate insulin secretion is controversial^24–26^ and has not been previously tested in isolated islets. Therefore, we tested if pharmacologic levels of CCK could directly potentiate insulin secretion *ex vivo*. We performed static GSIS studies in the presence of 100 nM^21^ of sulfated (pGlu-Gln)-CCK-8, a stable CCK analog peptide^27^, at either low or high glucose. Contrary to our expectations, we found that exogenous CCK did not affect GSIS in human or mouse islets (Fig. 5). We then tried a high concentration of (pGlu-Gln)-CCK-8, 1 µM. In a recent publication, Khan, *et al*. found that only at this 1 µM concentration of (pGlu-Gln)-CCK-8 was there a modest increase in GSIS in isolated mouse islets^28^. However, we were unable to replicate these findings in mouse islets and even at this high concentration (pGlu-Gln)-CCK-8 did not augment GSIS (Fig. 5C). As CCKR signaling can stimulate increases in intracellular calcium (Ca^2+^) in rat islets and other cell types^21,29,30^ and this could be the trigger for insulin secretion in a β-cell, we measured cytosolic Ca^2+^ in β-cells of isolated mouse islets before and after the addition of 1 µM CCK. We did not see a rapid or significant increase in intracellular Ca^2+^ levels in response to CCK as would be expected to trigger insulin secretion (Fig. 5D). Importantly, all cells increased cytosolic Ca^2+^ after 1 µM acetylcholine (ACh), assuring that β-cell Ca^2+^ signaling was not impaired^31^. We repeated our GSIS assay in a 2 hour static incubation in INS-1 cells and again did not see any augmentation of GSIS with 100 nM (pGlu-Gln)-CCK-8 treatment (Suppl. Fig. 3). To ensure that this was not an effect of the modified (pGlu-Gln)-CCK-8 peptide, we repeated the assay with sulfated CCK-8, a native CCK peptide^27^, and still did not stimulate insulin secretion in β-cells whether normalized to insulin content or DNA content (Suppl. Fig. 3). Collectively, our results show no evidence that CCK directly promotes insulin secretion.

## Discussion

The gut peptide hormone GLP-1 has been widely studied for its beneficial effects on β-cell insulin secretion and blood glucose control^6^. CCK has also been documented to stimulate insulin release in rodents^21,28^ and humans^26^. Here, we show that both hormones are secreted from mouse and human pancreatic islets but only GLP-1 acts locally within the islet to promote insulin release. Additionally, we demonstrate that while CCK expression is regulated by glucose, its receptor signaling is dispensable for GSIS. Our data highlight the critical role of paracrine GLP-1 within the islet to maintain glucose homeostasis. We find that CCK is expressed and dynamically regulated in human islets, but its paracrine role in islet function remains unknown.

GLP-1 produced by L cells in the gut is classically thought to act as an endocrine hormone on β-cells through the circulation. We and others have shown that islet GLP-1 is increased during conditions of islet-stress^2,4,14,15,32^, likely through increased pro-glucagon transcription and PC1/3 expression^9^. PC1/3 is highly expressed in β-cells because it is required for pro-insulin processing^33^. However, previous studies have shown that pancreatic α-cells contain the cellular machinery necessary to synthesize and secrete GLP-1, including PC1/3^2,3,13^ (and online dataset from Benner *et al*.^34^), suggesting that GLP-1 could also be produced in non-stressed islets. Based on these data, we^9^ and others^12,15^ have hypothesized that α-cell-derived GLP-1 acts on β-cell GLP-1R^9^. Here, GLP-1R antagonist markedly inhibited GSIS in islets from lean mice as well as from non-diabetic human donors, demonstrating that GLP-1 paracrine signaling pathways are already active in non-stressed β-cells (Fig. 2B). This suggests that there is a sufficient amount of GLP-1 present locally within the islet to activate its receptor on the neighboring β-cell. Indeed, we detected active GLP-1 being synthesized and released from both mouse and human islets, with an even greater amount in human islets than previously appreciated (Figs. 1 and 2A).

Previous studies have similarly suggested a paracrine signaling between α and β-cells where α-cell products directly regulate β-cell function^35^. A recent study showed that β-cell function depends on a local intra-islet glucagon signaling in mouse islets^36^. Furthermore, it has been shown that β-cell GLP-1R is necessary for glucose homeostasis and is dependent on *Gcg* peptides being produced in the islet^10^. Our study supports these findings and provides new evidence of α-cell-derived GLP-1 acting within the islet to promote β-cell function. Interestingly, glucagon has been reported as a relative low-potency agonist of GLP-1 receptor^37^, including in primary rodent β-cells^36,38^. Although our present data show that human islets produce and release much more GLP-1 in comparison to lean mouse, we cannot account for the possibility that other α-cell-derived peptides (i.e. glucagon) are also acting on β-cell GLP-1R^36,38^. Nevertheless, there is a limited understanding of this local signaling *in vivo* and more research is needed to confirm the relative role of α-to-β-cell crosstalk in regulating glucose homeostasis in humans.

In the present study, we also addressed whether pancreatic CCK might influence islet function in a paracrine manner. Since CCK has been shown to stimulate insulin secretion in rat islets^21^ and humans^26^, we predicted that islet-derived CCK would affect β-cell insulin secretion. However, blocking CCK receptors did not decrease GSIS, suggesting that intra-islet CCK does not potentiate insulin release via its receptors. Moreover, there was no insulinotropic action of CCK analog peptide in isolated islets. This result was a bit surprising, however most of the studies demonstrating a direct effect of CCK on GSIS were performed in rat islets^21,39^. Several other *in vivo* studies in mice and humans failed to demonstrate enhanced GSIS with CCK administration^25,27,40^. While the exact experimental conditions (CCK concentration, incubation methods) differ in much of the literature, we directly tested conditions previously used to demonstrate that CCK peptide at 1 μM enhanced GSIS in mouse islets and could not replicate those findings (Fig. 5C)^28^. Interestingly, our live-cell imaging results suggest that CCK peptide does not activate robust increases in intracellular Ca^2+^ in mouse β-cells as it does in pancreatic acinar cells^30^ or vagal afferent neurons^29^ and in rat islet^21^, and this lack of an effect on calcium influx may explain the inability to stimulate insulin secretion. In humans, both CCK receptor antagonists and CCK peptides did not affect insulin release *ex vivo* (Figs. 4B and 5B) or *in vivo*^41,42^, suggesting that CCK does not act as physiological incretin hormone. However, as CCK receptors are also expressed in other tissues, the peptide may be involved in the neural regulation of insulin secretion^43^, reduction of hepatic glucose production^11^ and control of food intake^27^. CCK may also play a role in other regulated insulin secretion, such as amino acid-stimulated insulin secretion^44^ or the enhancement of GSIS by GLP-1^45^. These beneficial effects, along with the impact of CCK on β-cell survival^4,5,46^, still make CCK a strong therapeutic candidate with the potential to treat obesity and type 2 diabetes^47^.

With increasing data indicate that classical gut hormones are produced locally in the islet^4,13,48,49^, many questions remain to be answered. For example, the molecular signals that turn on GLP-1/CCK expression in islet-cells remain to be fully elucidated. Also, a more detailed analysis of GLP-1/CCK-expressing cells in both mouse and human islets is warranted given there are several species differences. Both hormones act not only locally, but also systemically, increasing the complexity of understanding their overall impact on glucose homeostasis and beta-cell mass regulation. We have previously shown that overexpressing CCK in mouse β-cells does not change exocrine pancreas histology, nor systemic circulation of CCK peptide, suggesting that islet-derived hormone likely does not contribute significantly to systemic levels^46^. Similarly, recent studies showed that pancreatic GLP-1 does not necessarily change systemic GLP-1 circulating levels in mice^10,50,51^. These findings enhance our understanding on the biology of incretins and may open new possibilities for therapeutic innervations, including directed therapies to activate local pathways and avoid systemic side effects.

Overall, our results point to a physiological significance of paracrine GLP-1 but not CCK signaling in promoting glucose-stimulated insulin secretion in both mouse and human islets, suggesting that α to β-cell communication through GLP-1 receptor signaling is critical in the control of islet function and glucose homeostasis. Paracrine signaling of CCK is likely more important in β-cell survival pathways than in β-cell function and may be dynamically regulated by hyperglycemia.

## Methods

### Animals, islet isolation and culture

Animal care and experimental procedures were performed with approval from the Institutional Animal Care and Use Committee from the University of Wisconsin (protocol M005210) and William S. Middleton Memorial Veterans Affairs (protocol DD0001) to meet acceptable standards of humane animal care. All experiments were carried out in accordance with their guidelines and regulations. All animals used in this study were housed in facilities with a standard light-dark cycle and fed ad libitum. Pancreatic mouse islets were isolated from male 12–16 week old C57BL/6J and B6.Cg-*Lep*^*ob*^/J (*ob*/*ob*) (Jackson Laboratory, ME) as described previously^52^. Briefly, islets were isolated by collagenase digestion and Histopaque gradient (Sigma, #10771). Then, islets were handpicked and cultured at 37 °C and 5% CO_2_ in RPMI 1640 media (Thermo Fisher Scientific, #11875093) containing 11 mM glucose and supplemented with 5 g/l BSA fraction V (Roche, #107351080001), 100 units/ml penicillin and 100 μg/ml streptomycin (1% P/S) (Thermo Fisher Scientific).

### Human islets

Human islets were obtained through the Integrated Islet Distribution Program (https://iidp.coh.org/) (Suppl. Table 1). Upon arrival, islets were handpicked and then cultured in RPMI 1640 media (Thermo Fisher Scientific, #11879020) containing 8 mM glucose and supplemented as described above. Islets were cultured up to 7 days and the media was renewed every other day.

### mRNA levels

Gene expression analysis was performed as previously described^4,46^. RNA was isolated using either TRIzol or the QIAGEN RNeasy mini kit according to the manufacturer’s instructions. cDNA was prepared using Applied Biosystems High Capacity cDNA synthesis kit, then analyzed by quantitative PCR (qPCR) using Power SYBR Master Mix (Life Technologies). All transcripts were normalized to β-actin. mRNA levels are shown as −ΔC_T_ (CT[housing keeping] − CT[interest gene]). Fold changes were calculated relative to controls.

### Islet immunohistochemistry

Islets were dispersed at 37 °C using 0.25% Trypsin-EDTA (Thermo Fisher Scientific, #25200056), plated on Poly-L-lysine pre-coated glass coverslips and fixed with 10% formalin. Samples were permeabilized, blocked (Dako, #X0909) and then incubated with rabbit anti-glucagon (Santa Cruz, 1:200 – discontinued) and mouse anti-GLP-1 (7–36) amide (Abcam #ab26278, 1:200) antibodies. Abcam #ab26278 anti-GLP-1 (7–36) amide antibody recognizes the amidated C-terminus of the GLP-1(7–36) peptide and shows no cross-reactivity with non-amidated GLP-1, GLP-2, glucagon, or GIP^53^. Anti-rabbit Alexa Fluor 594 (1:400) and anti-mouse Alexa Fluor 488 (1:400) were used as secondary antibodies. Imaging was performed using an EVOS FL microscope. Manual scoring of images for co-localized glucagon, GLP-1, and DAPI (Dako) was performed using ImageJ (NIH) or Photoshop (Adobe) of, at least, 9 randomly chosen fields per treatment group for each replicate. DAPI staining was used as a nuclear marker while glucagon and GLP-1 were set as cytoplasmic stains.

### Hormone secretion and assay

Islet GLP-1 and CCK secretion were previously described^4^. Briefly, islets were incubated at a density of 1 islet per 10 µl of media. For GLP-1 secretion studies, the DPP4 inhibitor (Millipore) was added to the media to prevent GLP-1 degradation^54^. A membrane permeable cAMP analog (8-CPT-cAMP, referred to as cAMP) was used to stimulate CCK secretion as previously described^4^. After incubation, media was collected, centrifuged at 1000 rpm for 5 min at 4 °C and supernatant stored at −80 °C until further analysis. GSIS was carried out as described previously^52^. Briefly, 45–60 islets were pre-incubated for 30 minutes in 0.5 mM glucose at 37 °C. Batches of 5 islets were then incubated for 1 hour at 37 °C in 24-well plates with different glucose concentrations and chemical compounds such as, exendin-(9–39) (Ex9) (Tocris Bioscience, #2081), proglumide (Tocris Bioscience, #1478), sulfated CCK-8 peptide (Cayman Chemical, #23371), and sulfated (pGlu-Gln)-CCK-8 (American Peptide). More details about glucose concentrations and compounds utilized can be found in each figure legend.

GLP-1 was measured using an Active GLP-1 ELISA (Millipore, #EGLP-35K). This ELISA does not detect any other forms of GLP-1, GLP-2, or glucagon (manufacturer’s product information). Sulfated CCK levels were measured by radioimmunoassay with no cross-reactivity to the highly similar gastrin peptides (Alpco Diagnostics, now discontinued) as described^22^. Insulin was measured using an in-house insulin ELISA^55^, and QuantiFluor® dsDNA System (Promega, #E2670) was used to measure islet DNA.

### Live-cell imaging

Lean mouse islets were dispersed into small cell clusters using trypsin and gentle pipetting. Clusters were plated onto 35 mm culture dishes with cover glass bottom (Word Precision Instruments, #FD35) and cultured at 37 °C in the presence of 5% CO_2_ with RPMI 1640 medium containing 8 mM glucose, supplemented with 10% fetal bovine serum (FBS) and 1% P/S. For measurements of cytosolic Ca^2+^, cell clusters were pre-incubated with 2.5 μM FuraRed (Molecular Probes, #F3020) in RPMI media for 45 min at 37 °C. At the end of the pre-incubation, the cells were washed and placed into a bicarbonate-buffered Krebs solution containing 120 mM NaCl, 4.8 mM KCl, 2.5 mM CaCl_2_, 1.2 mM MgCl_2_, 24 mM NaHCO_3_, 1 g/l BSA, 10 mM HEPES, and 8 mM glucose. Fluorescence imaging of cytosolic Ca^2+^ was carried out as previously described^38^ except for the 40X/1.30 Oil S Fluor objective (Nikon Instruments). The excitation (ET430/20x and ET500/20x, ET type, Chroma Technology Corporation) and emission (650/60 m) filters (BrightLine type, Semrock) were used in combination with an FF444/521/608-Di01 dichroic beamsplitter (Semrock) and reported as an excitation ratio (R430/500).

### Statistical analysis

Results are presented as mean ± standard error of the mean (SEM). Unless otherwise stated, statistical significance was determined by two-way ANOVA and Bonferroni’s post-hoc test, as appropriate, using GraphPad Prism 8. Differences were considered significant when P ≤ 0.05.

## Supplementary information

Supplementary Material.

## Data Availability

Materials, data, and associated protocols will be made available upon request.
