# Supplementary material for: Intra-islet GLP-1, but not CCK, is necessary for β-cell function in mouse and human islets
Source: Sci Rep. 2020 Feb 18;10:2823. doi: 10.1038/s41598-020-59799-2 (PMC7028949; doi:10.1038/s41598-020-59799-2)
Supplement: Supplementary file 1 — Supplementary Material. [file 41598_2020_59799_MOESM1_ESM.docx]

**Intra-islet GLP-1, but not CCK, is necessary for β-cell function in mouse and human islets**

Arnaldo Henrique de Souza^1^, Jiayin Tang^1^, Amanjot Kaur Yadev^1^, Samuel Saghafi^1^, Carly R. Kibbe^1,2^, Amelia K. Linnemann^1,3^, Matthew J. Merrins^1,4^, and Dawn Belt Davis^1,4^*

**Supplementary material**

**Supplemental Figure 1**

**Suppl. Figure 1.** Representative images of co-localization of GLP-1 (7-37) (green) (A), glucagon (red) (B), or both merged with DAPI (blue) (C) in intact mouse islets. Scale bar, 200 µm.

**Supplemental Figure 2**

**Suppl. Figure 2. Correlation between donor BMI and CCK, CCKAR, or CCKBR gene expression in human islets**. Pearson correlation coefficient and R-squared (R^2^) of donor body mass index (BMI) and islet *CCK*, *CCKAR*, or *CCKBR* mRNA level. Data are from 30 (CCK) and 5 individual donors (CCKAR and CCKBR).

**Supplemental Figure 3**

**Suppl. Figure 3. Both (pGlu-Gln)-CCK-8 and CCK-8 peptides do not stimulate GSIS in rat β-cells**. Insulin secreted by INS-1 832/13 cells after 2h incubation at 3 or 15 mM glucose (3G and 15G, respectively) in the presence of 100 nM sulfated pGlu-Gln-CCK-8 or sulfated CCK-8. Insulin was normalized to insulin (**A**) or DNA (**B**) content. Significance was tested by two-way ANOVA and Bonferroni’s post-hoc test. Data are mean ± SEM (n=2).

**Suppl. Table 1. Human islet donors**

Adapted from Hart NJ, Powers AC (2018) Progress, challenges, and suggestions for using human islets to understand islet biology and human diabetes. Diabetologia.

| **Islet preparation** | **1** | **2** | **3** | **4** | **5** | **6** | **7** | **8** | **9** |
| --- | --- | --- | --- | --- | --- | --- | --- | --- | --- |
| **MANDATORY INFORMATION** | | | | | | | | | |
| Unique identifier | SAMN08768783 | SAMN08773445 | SAMN08768972 | SAMN08619527 | SAMN08832066 | SAMN08934018 | SAMN09091256 | SAMN09370567 | SAMN09666985 |
| Donor age (years) | 59 | 57 | 27 | 28 | 58 | 43 | 45 | 32 | 49 |
| Donor sex (M/F) | F | F | M | M | M | F | M | M | M |
| Donor BMI (kg/m^2^) | 32.3 | 25.8 | 30 | 34.7 | 31.8 | 30.9 | 29.3 | 28.5 | 30.3 |
| Donor blood glucose control (mg/dL) | 203 | 147 | 108 | 157 | 154 | 158 | 155 | 146 | 138 |
| Origin/source of islets | IIDP | IIDP | IIDP | IIDP | IIDP | IIDP | IIDP | IIDP | IIDP |
| Islet isolation centre | UW-Madison | UW-Madison | UW-Madison | Scharp-Lacy | Scharp-Lacy | Scharp-Lacy | Scharp-Lacy | UW-Madison | Scharp-Lacy |
| Donor history of diabetes? Please select yes/no from drop down list | No | No | No | No | No | No | No | No | No |
| **RECOMMENDED INFORMATION** | | | | | | | | | |
| Donor cause of death | Cerebrovascular/stroke | Cerebrovascular/stroke | Head trauma | Head trauma | Cerebrovascular/stroke | Head trauma | Cerebrovascular/stroke | Head trauma | Anoxia |
| Estimated purity (%) | 80% | 90% | 95% | 95% | 90% | 90% | 90% | 95% | 85% |
| Estimated viability (%) | 98% | 98% | 99% | 95% | 95% | 95% | 95% | 91% | 95% |
| Total culture time (h) | 0 days 20 hours | 3 days 13 hours | 1 day 19 hours | 3 days 7 hours | 4 days 2 hours | 5 days 1 hour | 2 days 0 hour | 0 days 20 hours | 2 days 0 hours |

**Supplemental Methods**

*Cell Culture*

INS-1 832/13 cells, a rat β-cell line^1^, were cultured at 37°C and 5% CO_2_ in RPMI 1640 media supplemented with 10% fetal bovine serum, 1% penicillin/streptomycin, 10 mM HEPES, 2 mM L-glutamine, 1 mM sodium pyruvate, and 50 µM β-Mercaptoethanol.

*GSIS assay*

Cells were seeded in a 96-well plate 24h prior studies. Then, cells were washed with 3 mM glucose (3G) Krebs-Ringer Bicarbonate buffer (KRB) (mM) NaCl (120), KCl (4.8), CaCl_2_ (2.5), MgCl_2_ (1.2), NaHCO_3_ (24), and 1 g/l BSA followed by preincubation for 2 h at 37°C in 3G. Thereafter, cells were washed with 3G, prior to a 2 h incubation under low (3 mM) and high (15 mM) glucose in the presence of 100 nM sulfated (pGlu-Gln)-CCK-8 or sulfated CCK-8 (Cayman Chemical, #23371). After 2 h, medium was collected, centrifuged for 5 min 1000 rpm at 4°C and the supernatant stored at −20°C until analysis. Cells were lysed by sonication in TNE buffer (Tris 10 mM, NaCl 0.2 mM, EDTA 10 mM) and samples were stored at −20°C until analysis.

**References**

1 Hohmeier, H. E. *et al.* Isolation of INS-1-derived cell lines with robust ATP-sensitive K+ channel-dependent and -independent glucose-stimulated insulin secretion. *Diabetes* **49**, 424-430, doi:10.2337/diabetes.49.3.424 (2000).
